# The New York City Marathon: A systematic review of performance, participation, pacing, and health-related outcomes

**DOI:** 10.17179/excli2026-9354

**Published:** 2026-04-29

**Authors:** Beat Knechtle, Mabliny Thuany, Pantelis T. Nikolaidis, Pedro Forte, Luciano Bernardes Leite, Romuald Lepers, Daniela Chlíbková, Katja Weiss, Thomas Rosemann, Rodrigo L. Vancini, Sasa Duric

**Affiliations:** 1 Medbase St. Gallen Am Vadianplatz, St. Gallen, Switzerland; 2 Institute of Primary Care, University Hospital Zurich, Zurich, Switzerland; 3 Department of Sports, State University of Pará, Belém, Brazil; 4 School of Health and Caring Sciences, University of West Attica, Athens, Greece; 5 Department of Sports, Higher Institute of Educational Sciences of the Douro, Penafiel, Portugal; 6 Department of Sports Sciences, Instituto Politécnico de Bragança, Bragança, Portugal; 7 Research Center for Active Living and Wellbeing (Livewell), Instituto Politécnico de Bragança, Bragança, Portugal; 8 Department of Physical Education, Federal University of Viçosa, Viçosa, Brazil; 9 Université Bourgogne Europe, INSERM, CAPS UMR 1093, Dijon, France; 10 Centre of Sports Activities, Brno University of Technology, Brno, Czechia; 11 Center of Physical Education and Sports, Federal University of Espírito Santo, Vitória, Espírito Santo, Brazil; 12 Liberal Arts Department, American University of the Middle East, Egaila, Kuwait

**Keywords:** endurance running, environmental conditions, pacing, heart

## Abstract

The 'New York City Marathon' is one of the world's largest and most influential mass-participation marathons. Although numerous studies have examined performance trends, participation patterns, pacing behavior, environmental influences, and physiological aspects of runners in this event, no review has synthesized the evidence specific to this race. This study aimed to systematically summarize the scientific literature on the 'New York City Marathon'. A systematic search of Scopus, PubMed, Web of Science, Embase, and Cochrane was conducted using terms related to the 'New York City Marathon' to identify studies published up to February 2026. Eligible studies included runners of all ages, sexes, and performance levels, with no restrictions on publication date, topic, or study design. Extracted data included: (1) authors; (2) publication year; (3) study design; (4) sample characteristics; (5) variables assessed; and (6) main findings. Results were synthesized narratively by domain. Seventy-six publications met the inclusion criteria. Participation increased markedly over time, driven primarily by growth among women and age-group runners. While elite and competitive age-group performances improved in recent decades, mean finish times across the entire field increased by ~40 min since the 1970s, reflecting the democratization of marathon running. Ethiopian runners were the youngest and fastest. Peak performance occurred at 29.7 years in women and 34.8 years in men (1-year age intervals), and in the 30-34 and 35-39 age groups, respectively (5-year intervals). Approximately 10 % of runners experienced major injuries during training or the race that prevented starting or finishing. Higher training volumes increased injury risk, with foot, knee, and hip injuries most common, whereas adequate preparation reduced risk. Environmental conditions-particularly temperature-had a stronger influence on race times than course metrics. Performance declined with increasing temperature, especially among slower runners and among men aged 30-64 and women aged 40-64. Runners generally adopted a positive pacing strategy with a final spurt in the last segment (40-42.2 km). The fastest split occurred between 5-10 km and the slowest between 35-40 km, coinciding with the undulating terrain entering Central Park. Older athletes paced more evenly than younger athletes. Men showed a larger decrease in running speed from the fastest to the slowest splits than women (21.1 % vs. 16.7 %). Slower runners exhibited greater early-race deceleration but larger late-race speed increases, whereas faster runners maintained the most even pacing. Participation in the 'New York City Marathon' has grown substantially, driven by increased involvement of women and age-group runners. Although elite performance has improved, overall mean finish times have slowed due to broader participation. Ethiopian runners were the youngest and fastest, with peak performance occurring in the early to mid-30s. Injury prevalence was considerable, particularly with higher training volumes, though adequate preparation mitigated risk. Higher temperatures slowed performance, especially among slower runners. Pacing was predominantly positive, with older athletes pacing more evenly and faster runners showing the smallest performance decline. Future research should explore cardiovascular monitoring technologies-including real-time ECG streaming during the race-and assess the impact of innovations such as carbon-plated “supershoes” on performance and pacing.

See also the graphical abstract(Fig. 1).

## Introduction

The 'New York City Marathon' (also referred to as the *New York Marathon* and officially the *TCS New York City Marathon* after its title sponsor, Tata Consultancy Services) is held annually on the first Sunday in November in New York City. Organized since 1970 by the New York Road Runners (Burfoot, 2007[14]), it is, together with the 'Boston Marathon' and the 'Chicago Marathon', one of the largest and most influential running events in the United States of America. In 2006, these three races, along with the 'London Marathon' and the 'Berlin Marathon', formed the World Marathon Majors (Díaz et al., 2019[27]).

Participation has grown steadily over the decades. In 2016, the event recorded 51,394 finishers out of 51,999 starters (Dutch and Boswell, 2026[30]), and in 2019, a total of 53,639 runners completed the race. Apart from the 50^th^ edition of the 'Berlin Marathon' in 2024, which saw 53,994 finishers, the 'New York City Marathon' remains the marathon with the highest number of participants worldwide.

Originally a small event held entirely within Central Park and attracting only a few hundred runners, the 'New York City Marathon' expanded in 1976 to traverse all five boroughs of New York City in celebration of the United States Bicentennial (New York Road Runners, 2026[78]). This new course format proved highly successful and has been maintained ever since. The race has been canceled only twice in its history: in 2012, two days before the start, due to the aftermath of Hurricane Sandy, and in 2020 because of the COVID-19 pandemic. While the early editions took place in mid-September, the event was held at the end of October from 1976 to 1985. Since 1986, it has been scheduled for the first Sunday in November, with the exception of 1993 and 1995, when it was held on the second Sunday of the month (New York Road Runners, 2026[77]).

The 'New York City Marathon' traverses all five boroughs of New York City, incorporating multiple bridge crossings, rolling terrain, and a demanding finish through Central Park. The course features ⁓247 m of elevation gain and ⁓251 m of elevation loss, making world-record performances highly unlikely under current standards (Díaz et al., 2019[27]). Although this holds true for contemporary men's world-record attempts, historical world records have been set at the event, including those by Grete Waitz and Alberto Salazar. The scientific relevance of the race also has longstanding roots: in 1976, the New York Academy of Sciences hosted the first major scientific and medical conference dedicated to the marathon, titled *The Marathon: Physiological, Medical, Epidemiological, and Psychological Studies* (Costill, 2007[22]).

Despite its historical, cultural, and scientific importance, no systematic synthesis of research specifically focused on the 'New York City Marathon' has been conducted. Seminal work has established the marathon as a landmark event in endurance running and sports science-ranging from early physiological and medical investigations linked to the 1976 edition (Costill, 2007[22]) to more recent analyses of pacing and performance across the World Marathon Majors (Díaz et al., 2019[27])-yet the available literature remains fragmented and dispersed across broader marathon or multi-event comparisons. Moreover, the unique characteristics of the 'New York City Marathon'-including its demanding course profile, substantial elevation changes, environmental variability, and exceptionally large and heterogeneous participant base-distinguish it from other major marathons and limit the generalizability of findings derived from flatter, record-oriented courses (Díaz et al., 2019[27]).

Although its scale and complexity make it an ideal natural laboratory for studying performance, pacing strategies, physiological responses, and health-related outcomes in mass-participation endurance events, no comprehensive review has yet consolidated and critically appraised the body of research conducted in this specific context.

Despite the race's global prominence and extensive participation, the available research remains scattered across diverse domains, making it difficult for athletes, coaches, and researchers to access an integrated body of knowledge. The aim of this study was therefore to systematically collect and summarize all scientific literature pertaining to the 'New York City Marathon', providing a comprehensive resource for future preparation, performance optimization, and research development. A dedicated review is warranted to map existing evidence, identify methodological trends and limitations, and highlight underexplored dimensions of research related to this event. Such an approach will help guide future investigations and advance understanding within marathon and endurance sport science. We hypothesized that, despite heterogeneity in study designs and research themes, consistent patterns would emerge across key domains - including participation trends, performance, pacing behavior, aging effects, injury epidemiology, and environmental influences. Identifying these patterns would enable a comprehensive interpretation of how athletes perform and adapt within the specific context of the 'New York City Marathon'.

## Methods

### Protocol and registration

This systematic review was conducted according to the 2020 Preferred Reporting Items for Systematic Reviews and Meta-Analyses (PRISMA) statement (Page et al., 2021[87]). The protocol was registered with the International Prospective Register of Systematic Reviews (PROSPERO 2026 CRD420261278395).

### Search strategy and eligibility criteria

The databases Scopus, PubMed, Web of Science, Embase, and Cochrane were used to retrieve articles. The selected articles were related to the 'New York City Marathon' and were published up to February 2026 with no language restrictions. Non-English articles were **screened by abstract only**. The following search terms were used: ((New York City Marathon) OR (TCS New York City Marathon) OR (New York Marathon) OR (New York City race) OR (NYC Marathon)) AND (performance OR pacing OR injury OR physiology OR age OR heart). Table 1(Tab. 1) presents the eligibility criteria for studies included in this review.

The inclusion criteria encompassed original research studies focusing on different research topics related to runners participating in the 'New York City Marathon'. Studies were eligible regardless of participants' age, sex, or level of competitiveness, thereby including recreational, amateur, and elite athletes. Grey literature, book chapters, editorials, and conference proceedings were excluded. No restrictions were applied regarding publication date or language. Data extraction was performed by two authors (BK, MT) for all selected studies, considering: (1) authors; (2) year of publication; (3) study design; (4) sample characteristics; (5) variables assessed; and (6) main results. Studies were grouped by similarity and the results were reported. Inter-reviewer agreement for study selection was high (κ > 0.80). To ensure a critical synthesis, two investigators independently assessed the methodological quality of the included studies using the Newcastle-Ottawa Scale (NOS) for observational research, except for case studies. Discrepancies were resolved by a third investigator.

## Results

**Figure 2:**
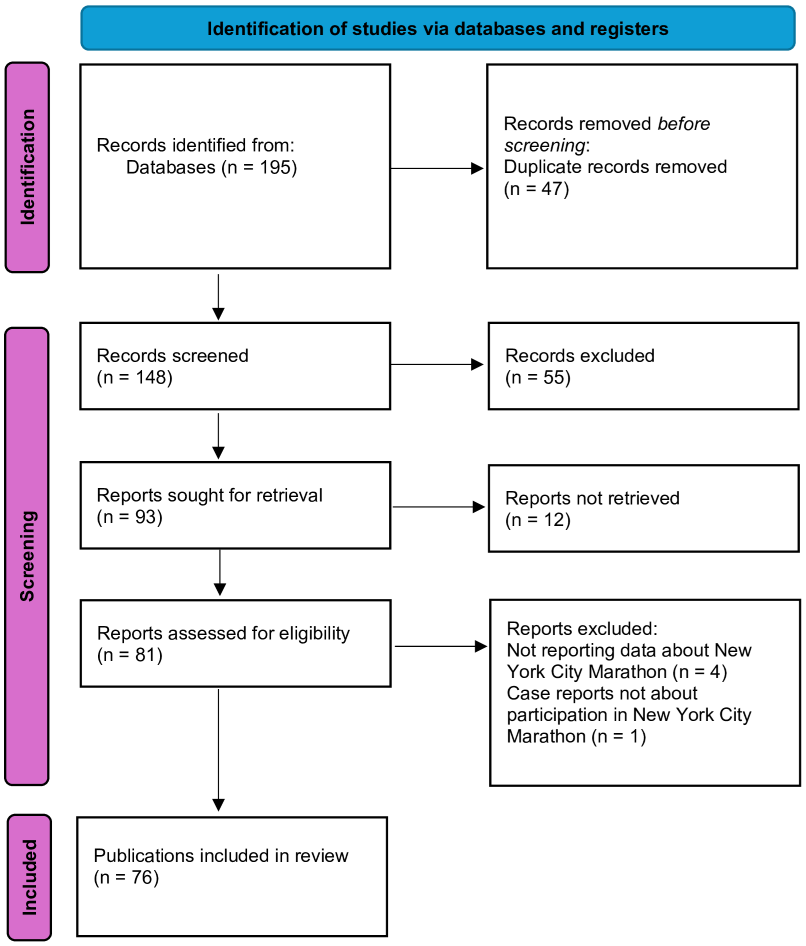
PRISMA flow diagram of the screening process

We identified 195 publications in total: 67 in Scopus, 63 in PUBMED, 37 in Web of Science, 25 in Embase, and 3 in Cochrane. Ultimately, 76 publications were included in the review. The PRISMA flowchart detailing the search and selection strategies is presented in Figure 2(Fig. 2). The results are organized into six main research domains: participation trends, performance and aging, nationality-related performance patterns, injury epidemiology, environmental influences, and pacing strategies.

### Participation and performance trends in the 'New York City Marathon'

Since the 1976 'New York City Marathon', the first edition to adopt the modern 'urban tour' format, marathon running has expanded rapidly worldwide. Growth has been especially pronounced among women and master runners (> 35 years) (Burfoot, 2007[14]). Overall participation in the 'New York City Marathon' has increased steadily (Duric et al., 2026[29]), with a disproportionately large rise among women (Nikolaidis et al., 2018[84]; Vitti et al., 2020[113]) and age-group athletes (Duric et al., 2026[29]; Vitti et al., 2020[113]). In recent decades, performance has improved among elite runners and among the top 50 master (age-group) athletes of both sexes (Jokl et al., 2004[46]; Garatachea et al., 2014[37]). At the same time, mean finishing times across the entire field have increased-reflecting the growing proportion of recreational (master, age-group) participants (Vitti et al., 2020[113]).

In the inaugural 1970 race, estimated finishing times ranged from ⁓3:00 h:min to 3:10 h:min, based on the performance distribution of the 55 finishers, all of whom were competitive runners. In contrast, recent editions report mean finishing times of ⁓4:40 h:min (Vitti et al., 2020[113]). Although participation trends consistently show substantial increases over time-particularly among women and older runners-many studies do not clearly differentiate between absolute numbers of finishers and proportional representation by sex or age group. Importantly, much of the observed growth appears to be driven by increases in total race capacity rather than shifts in relative participation alone.

#### Sex differences in participation and performance

Overall participation in the 'New York City Marathon' increased over time, with more men than women finishing the race (Duric et al., 2026[29]). However, the rise in participation was more pronounced among women, leading to a progressive decline in the men-to-women ratio (Nikolaidis et al., 2018[84]; Vitti et al., 2020[113]). Between 2006 and 2026, the total men-to-women ratio was 1.68. Compared to 2006 (n = 12,321), 21,441 women participated in 2016, i.e. an increase of +74.0 %, whereas the corresponding scores in men were 25,558, 29,829 and +16.7 %, and in both women and men 37,879, 51,270 and +35.4 %. A sex × calendar year association was shown, where the men-to-women ratio was the smallest in 2016 (1.39) and the largest in 2007 (2.08) (Nikolaidis et al., 2018[84]).

The total number of finishers from 1970 to 2017 reached 1,174,331, of which 349,145 were women and 825,186 were men. The 1970s showed the lowest number of finishers (25,299), the 2000's the highest (351,162) for now, with the 2010's counting 344,126 finishers already. The number of both women and men finishers increased continuously across the years, this increase being more pronounced in women, which was highlighted by the decrease of the men-to-women ratio across the years. However, the number of female finishers never exceeded the number of male finishers (Vitti et al., 2020[113]).

Sex differences in marathon performance have been examined using data exclusively from the 'New York City Marathon' (Nikolaidis et al., 2018[84]; Vitti et al., 2020[113]; Hunter and Stevens, 2013[44]) as well as datasets combining 'New York City Marathon' with multiple marathon events (Hunter et al., 2011[45]). Across studies, men were generally faster than women among both elite (Vitti et al., 2020[113]; Hunter et al., 2011[45]) and master (recreational, age-group) runners (Vitti et al., 2020[113]; Nikolaidis et al., 2018[84]; Ahmadyar et al., 2015[1]).

The magnitude of the sex difference in performance varied with age and race time (Hunter and Stevens, 2013[44]). Among the top finishers, the sex difference in running speed increased from the 1^st^ to the 10^th^ place due to a greater relative decline in running speed among women compared to men (Hunter and Stevens, 2013[44]). This sex difference among 1^st^-place finishers was 19.9±7.7 % in 1980-1985 and declined to 12.6±4.0 % by 2006-2010 (Hunter and Stevens, 2013[44]).

The sex difference also widened with increasing age but decreased across calendar years, particularly in older age groups (Hunter and Stevens, 2013[44]). The relative sex difference in running speed of the 1^st^ finishers progressively increased with age with the least sex difference within the 30- to 34-years old age group (11.8±1.1 %) and the greatest within the 75- to 79-years-olds (24.3±11.4 %). Over time, the number of female finishers increased relative to male finishers, especially among older runners (Hunter and Stevens, 2013[44]).

Approximately 34 % of the sex difference in running speed among age group winners was explained by the men-to-women finisher ratio within each age category (Hunter and Stevens, 2013[44]), indicating that participation depth contributes meaningfully to the observed sex-based performance gaps.

#### The evolution of master runners

Participation among master runners (35 years and older) has increased markedly in recent decades, both globally and within the 'New York City Marathon' (Duric et al., 2026[29]; Jokl et al., 2004[46]; Vitti et al., 2020[113]; Lepers and Cattagni, 2012[59]). Growth has been especially pronounced in the master age groups compared with younger runners (Jokl et al., 2004[46]), and the increase has been greater among women than men (Lepers and Cattagni, 2012[59]). Overall, the number of master runners has expanded at a faster rate than that of their younger counterparts (Jokl et al., 2004[46]).

Regarding participation among age-group (master) runners, participation was lowest in the youngest (< 20 years) and oldest (75+ years) age groups (Duric et al., 2026[29]). Earlier studies reported that most female participants were in the 30-34 years age group and most male participants in the 40-44 years age group (Nikolaidis et al., 2018[84]), whereas more recent analyses identified the 40-44 years age group as the most represented for both sexes (Duric et al., 2026[29]).

Among very old master runners, participation increased and performance improved in both women and men competing in the 75-79 years to 95-99 years age groups, with the greatest increases in participation and the largest performance improvements occurring in the 75-79 years age group (Ahmadyar et al., 2015[1]).

Performance trends also showed notable improvements among master athletes. Male and female master runners continued to improve their race times relative to younger athletes (Jokl et al., 2004[46]). Race times for the top 50 male and female finishers improved more in the master groups than in younger age groups (Jokl et al., 2004[46]). Significant decreases in race times were observed for male runners older than 64 years and for female runners older than 44 years (Lepers and Cattagni, 2012[59]). Although sex differences in race times decreased over the years, they remained relatively stable across age groups in recent editions (Lepers and Cattagni, 2012[59]).

#### The aspect of nationality in the 'New York City Marathon'

Nationality has been examined in several studies focusing on participation and performance in the 'New York City Marathon' (Duric et al., 2026[29]; Vitti et al., 2020[113]; Knechtle et al., 2017[48]; Aschmann et al., 2018[3]). Across editions, the fastest average race times were predominantly achieved by both female and male runners from Kenya and Ethiopia (Duric et al., 2026[29]). Among the nationalities most frequently represented among top finishers, Ethiopian runners tended to be younger (Vitti et al., 2020[113]; Aschmann et al., 2018[3]) and achieved faster finishing times (Vitti et al., 2020[113]; Knechtle et al., 2017[48]). In contrast, German (Vitti et al., 2020[113]) and Japanese runners (Aschmann et al., 2018[3]) were the oldest, and Japanese runners were consistently the slowest among the major national groups analyzed (Vitti et al., 2020[113]; Aschmann et al., 2018[3]). Overall, the fastest and youngest runners originated from East Africa (Duric et al., 2026[29]).

Nationality-specific patterns also varied by age. Among runners younger than 20 years, stronger performances were observed among European athletes, particularly those from Poland, Switzerland, and Italy (Duric et al., 2026[29]). In age groups of 50 years and older, the best average race times were increasingly recorded by runners from the USA, Japan, Germany, and Switzerland (Duric et al., 2026[29]), suggesting that nationality-related performance differences shift with age and demographic representation.

### Age aspects in the 'New York City Marathon'

Age is a key determinant of marathon performance in the 'New York City Marathon' (Santos-Lozano et al., 2015[91]). Across editions, male participants were generally older than female participants (Nikolaidis et al., 2018[84]; Vitti et al., 2020[113]). Several studies have examined age-related dimensions of performance, including the age of peak performance (Nikolaidis et al., 2018[84]; Vitti et al., 2020[113]; Lara et al., 2014[57]), the age-related performance decline (Lara et al., 2014[57]; Zavorsky et al., 2017[122]), the broader effects of aging on marathon outcomes (Santos-Lozano et al., 2015[91]), and the characteristics of age-group (master) runners (Ahmadyar et al., 2015[1]; Connick et al., 2015[21]).

#### The age of peak performance

The age of peak marathon performance has been examined using data exclusively from the 'New York City Marathon' (Nikolaidis et al., 2018[84]; Vitti et al., 2020[113]; Lara et al., 2014[57]) as well as combined datasets including other major marathons (Hunter et al., 2011[44]; Zavorsky et al., 2017[122]). While one study reported no sex difference in the age of peak performance in the 'New York City Marathon' (Hunter et al., 2011[44]), others identified clear sex-specific patterns. The relationship between age and marathon performance followed a U-shaped curve (Lara et al., 2014[57]), with the fastest performances occurring between 25 and 34 years of age. Across World Marathon Majors, overall champion males averaged 28.3 years and champion females 30.8 years (Zavorsky et al., 2017[122]).

More detailed analyses of 'New York City Marathon' data showed that peak performance occurred at 29.7 years for women and 34.8 years for men when age was analyzed in 1-year intervals, and in the 30-34 and 35-39 age groups, respectively, when age was analyzed in 5-year intervals (Nikolaidis et al., 2018[84]). Another study reported the fastest race times at 27 years for men and 29 years for women (Lara et al., 2014[57]). Before reaching these ages, race time increased by 4.4±4.0 % per year in men and 4.4±4.3 % per year in women. After these ages, race time increased by 2.4±8.1 % per year in men and 2.5±9.9 % per year in women (Lara et al., 2014[57]).

The sex difference in race time remained stable at ⁓18.7±3.1 % between ages 18 and 57 years, after which the sex difference progressively increased with advancing age (Lara et al., 2014[57]).

#### Age-related performance decline in the 'New York City Marathon'

When the impact of aging on marathon performance was examined in both non-elite and elite runners, the overall effect of aging on performance was comparable between sexes. However, aging affected the fastest and slowest runners differently, and the magnitude of sex differences was greater among slower runners than among the fastest performers (Lara et al., 2014[57]).

From 35 to 74 years of age, female age group winners exhibited a faster yearly decline in race times compared with male age group winners (Zavorsky et al., 2017[122]). The decline in performance across these ages was nearly linear, with female winners showing a 27-second greater annual slowdown than their male counterparts (Zavorsky et al., 2017[122]).

#### Master marathoners in the 'New York City Marathon'

In recent decades, the number of master marathoners competing in the 'New York City Marathon' has increased substantially (Ahmadyar et al., 2015[1]). Among runners older than 75 years, participation in both women and men remained stable, and the fastest athletes in these age groups became slower over time (Ahmadyar et al., 2015[1]). Performance followed a curvilinear relationship with age, with the negative effect of aging becoming progressively more pronounced in older runners (Connick et al., 2015[21]).

Relative age effects were also evident. Within master age groups, relatively older male athletes (*i.e.,* those at the upper end of each 5-year age band) were competitively disadvantaged compared with younger athletes in the same category once they exceeded 50 years of age. Similarly, relatively older female athletes were disadvantaged from age 40 years onward (Connick et al., 2015[21]). In contrast, relative age effects were also observed in the 20-24 years age group, consistent with the broader pattern that marathon performance improves until peak performance is typically reached in the 25-29 years age range (Connick et al., 2015[21]).

### Running-related injuries in the 'New York City Marathon'

Several studies have examined running-related injuries occurring during training for, or participation in the 'New York City Marathon' (Caselli and Longobardi, 1997[15]; Toresdahl et al., 2020[110], 2022[108], 2023[111], 2025[109]; McGrath et al., 2024[71]). Reported injury prevalence varied widely across studies, largely due to differences in injury definitions, assessment methods, and observation periods. Approximately 9.5 % of runners experienced major injuries during training or the race that prevented them from starting or finishing (Toresdahl et al., 2022[108]), while an additional ⁓49.2 % sustained minor injuries that interfered with training and/or affected race performance (Toresdahl et al., 2022[108]). The prevalence of running-related overuse injuries during marathon preparation ranged from ⁓36.1 % (Toresdahl et al., 2025[109]) to ⁓38.4 % (McGrath et al., 2024[71]), and up to ⁓40 % in some cohorts (Toresdahl et al., 2023[111]).

Higher training volumes were consistently associated with increased injury incidence among runners preparing for the race (Caselli and Longobardi, 1997[15]; Toresdahl et al., 2023[111]). Foot, knee, and hip injuries were the most common during training (McGrath et al., 2024[71]). During the marathon itself, ⁓14.1 % (McGrath et al., 2024[71]) to ⁓16 % (Toresdahl et al., 2023[111]) of runners reported an injury. Injuries were particularly common among first-time marathon runners (Toresdahl et al., 2020[110]; Toresdahl et al., 2022[108]). In this group, the incidence of major injury was ⁓8.9 %, and minor injury was at ⁓48.5 % (Toresdahl et al., 2020[110]).

The most frequently reported race-day injuries included corns, calluses, blisters, muscle cramps, acute knee and ankle injuries, plantar fasciitis, and metatarsalgia (Caselli and Longobardi, 1997[15]). Knee, thigh, and foot injuries were also highly prevalent during the race (McGrath et al., 2024[71]). Overall injury prevalence reached ⁓42.6 % in one study (McGrath et al., 2024[71]). The most commonly affected tissue types were muscle, tendon/fascia, and bursa (McGrath et al., 2024[71]), with hamstring injuries showing the highest diagnostic prevalence at ⁓6.7 % (McGrath et al., 2024[71]).

Pre-race preparation appeared to influence injury risk. Runners who had completed a half-marathon prior to the study were less likely to report injuries (Toresdahl et al., 2022[108]). Those averaging fewer than four training runs per week were less likely to sustain injuries than those training four or more times per week (Toresdahl et al., 2022[108]). Additionally, greater longest-run distance during training was inversely associated with race-day injury incidence (Toresdahl et al., 2022[108]).

Strength-training interventions have also been evaluated for injury prevention. Programs targeting hip abductors and related muscle groups were associated with a 64 % lower risk of running-related overuse injury (Toresdahl et al., 2025[109]). However, in another study of first-time marathoners, a self-directed strength-training program did not reduce the incidence of overuse injuries leading to marathon non-completion (Toresdahl et al., 2020[110]).

### Medical aspects

A range of medical domains has been investigated in runners participating in the 'New York City Marathon'. Studies have examined cardiovascular responses and adaptations to marathon running (Augustine et al., 2021[4]; Bonetti et al., 1995[8]; Cerioli et al., 1995[19]), urogenital function (Milvy et al., 1981[72]), menstrual cycle-related considerations in female runners (Shangold and Levine, 1982[98]), gastrointestinal symptoms and disturbances (Moses et al., 1991[74]), and pulmonary function before and after the race (Aust, 2005[5]). Together, these studies highlight the broad physiological demands of marathon running and the diverse organ systems affected during preparation and competition.

#### Cardiovascular and metabolic responses

Several studies have examined changes in plasma lipids and cardiovascular function associated with participation in the 'New York City Marathon' (Augustine et al., 2021[4]; Bonetti et al., 1995[8]; Cerioli et al., 1995[19]). Marathon running induced notable alterations in lipoprotein (a), although findings differed depending on training status. In trained marathoners, lipoprotein (a) increased significantly one month after the race-during a detraining period-compared with both baseline and immediate post-race values (Cerioli et al., 1995[19]). In contrast, among untrained individuals, lipoprotein (a) decreased significantly beginning 24 hours after the race and remained below baseline levels for up to 72 hours (Bonetti et al., 1995[8]).

Cardiac adaptations and potential sex differences in central hemodynamics have also been investigated. No sex differences were observed in left-ventricular longitudinal strain (LS), circumferential strain (CS), area strain (AS), or radial strain (RS) (Augustine et al., 2021[4]). However, female marathoners exhibited a lower ratio of arterial elastance (Ea) to ventricular elastance (Elv)-a global index of ventricular-vascular coupling-compared with male marathoners (Augustine et al., 2021[4]), suggesting subtle sex-specific differences in cardiovascular load and efficiency during endurance exercise.

#### Urogenital system

One study investigated the prevalence of urolithiasis among male participants in the 'New York City Marathon' (Milvy et al., 1981[72]). The prevalence of kidney stones was substantially higher in marathon runners compared with matched control populations-five-fold higher in men younger than 45 years and three-fold higher in those aged 45 to 64 years (Milvy et al., 1981[72]). These findings suggest that long-term endurance training and repeated marathon participation may be associated with an elevated risk of urolithiasis in male athletes.

#### Pulmonary system

One study evaluated the impact of asthma management during pre-race preparation in endurance athletes with physician-diagnosed asthma (Aust, 2005[5]). The author examined runners preparing for the 'New York City Marathon' who were treated with a combination inhaled corticosteroid and long-acting β₂-agonist (budesonide and formoterol fumarate dihydrate; Symbicort^®^). The analysis focused on whether optimized asthma therapy during the preparatory phase influenced respiratory symptoms, perceived exertion, and overall readiness for competition.

#### Effect of marathon training on menstrual function

In one study examining menstrual function during marathon preparation for the 'New York City Marathon', the incidence of oligomenorrhea or amenorrhea increased from ⁓19 % prior to training to 24 % during the training period (Shangold and Levine, 1982[98]). The reported prevalence of infertility among participants was 10 % (Shangold and Levine, 1982[98]). Notably, among women who had regular menstrual cycles before initiating marathon training, ⁓93 % maintained regular menses throughout the training program (Shangold and Levine, 1982[98]).

#### Effect of cimetidine on marathon-associated gastrointestinal symptoms and bleeding

One study evaluated whether cimetidine administration influenced gastrointestinal symptoms or bleeding in participants of the 'New York City Marathon' (Moses et al., 1991[74]). The investigators found that cimetidine had no significant effect on occult gastrointestinal blood loss, as assessed by stool Hemoccult (HO) cards and Hemoquant (HQ) measurements (Moses et al., 1991[74]).

### Environmental conditions and race performance

Several studies have examined how environmental conditions influence running performance in the 'New York City Marathon'. Some analyses focused exclusively on this event (Knechtle et al., 2021[51]; Gasparetto and Nesseler, 2020[38]; Weiss et al., 2022[117][116]), whereas others combined 'New York City Marathon' data with results and meteorological records from additional international marathons (Maffetone et al., 2017[67]; El Helou et al., 2012[31]; Ely et al., 2007[32]; Llewellyn and Maceri, 2025[65]). In a comparative study evaluating race-course characteristics alongside environmental variables such as temperature, humidity, and altitude, weather conditions-rather than course metrics-emerged as the primary determinant of performance variation (Maffetone et al., 2017[67]). Because these analyses were conducted within a consistent race context, observed fluctuations in finishing times largely reflect environmental variability rather than differences in course profile.

Across studies, a wide range of meteorological variables were investigated, including temperature (Knechtle et al., 2021[51]; Weiss et al., 2022[117][116]; El Helou et al., 2012[31]; Ely et al., 2007[32]; Llewellyn and Maceri, 2025[65]), wet-bulb globe temperature (WBGT) (Gasparetto and Nesseler, 2020[38]), universal thermal climate index (UTCI) (Gasparetto and Nesseler, 2020[38]), humidity (Knechtle et al., 2021[51]; Weiss et al., 2022[117][116]; El Helou et al., 2012[31]), dew point (El Helou et al., 2012[31]), precipitation (Weiss et al., 2022[116]), sunshine duration (Weiss et al., 2022[117][116]), cloud cover (Weiss et al., 2022[116]), wind (Knechtle et al., 2021[51]), and atmospheric pressure (Weiss et al., 2022[117][116]; El Helou et al., 2012[31]).

#### Temperature

Temperature consistently emerged as the most influential environmental factor affecting marathon performance (Knechtle et al., 2021[51]; Gasparetto and Nesseler, 2020[38]; Weiss et al., 2022[117][116]; El Helou et al., 2012[31]; Ely et al., 2007[32]; Llewellyn and Maceri, 2025[65]). In contrast, other meteorological variables-such as humidity, dew point, and atmospheric pressure-showed no meaningful association with performance outcomes in most analyses (Weiss et al., 2022[116]; El Helou et al., 2012[31]). Across studies, a common pattern was observed where marathon race times slowed progressively as ambient temperature increased (Knechtle et al., 2021[51]; Weiss et al., 2022[117][116]; El Helou et al., 2012[31]; Ely et al., 2007[32]; Llewellyn and Maceri, 2025[65]). One investigation reported that high temperatures were associated with an average performance decrement of ⁓8 min, representing the largest single environmental effect identified (Knechtle et al., 2021[51]). Age also appeared to modulate the temperature-performance relationship. Running speed declined with rising temperatures among athletes aged 20-59 years, with particularly pronounced effects in men aged 30-64 years and women aged 40-64 years (Weiss et al., 2022[116]). Similarly, high temperatures disproportionately slowed finishers in these same age groups, whereas runners aged 70 years and older did not exhibit an increased sensitivity to heat stress (Knechtle et al., 2021[51]).

#### Humidity

Humidity appears to exert a small but measurable influence on marathon performance in the 'New York City Marathon'. Among elite runners (top-ten finishers), running speed was positively associated with higher humidity levels, indicating that these athletes tended to run faster as humidity increased (Weiss et al., 2022[117]). Similar patterns were observed in age group competitors, with higher humidity corresponding to faster running speeds in both women and men (Weiss et al., 2022[116]). However, the impact of elevated humidity on race time was not uniform across age groups where the slowing effect of high humidity was significantly greater in men aged 40-59 years and women aged 25-65 years (Knechtle et al., 2021[51]).

#### Wind

Wind also appeared to influence marathon performance, with slower race times observed on days characterized by low wind speeds (Knechtle et al., 2021[51]). The effect was not uniform across sexes where men were significantly more affected by variations in wind speed than women (Knechtle et al., 2021[51]). Also, age was of importance where an inverse association between race time and high wind speed was pronounced in finishers with younger age and less strong in finishers in age groups 40 years and older (Knechtle et al., 2021[51]).

#### Sunshine duration

Sunshine duration appeared to have a small but measurable influence on race performance in the 'New York City Marathon'. Among elite runners, a weak positive correlation was observed, indicating that these athletes tended to run faster with increasing sunshine duration (Weiss et al., 2022[117]). In contrast, sunshine showed no meaningful association with performance among age-group runners (Weiss et al., 2022[116]).

#### Performance level

The influence of environmental conditions on marathon outcomes varies according to runners' performance level (Gasparetto and Nesseler, 2020[38]; Weiss et al., 2022[117]; Ely et al., 2007[32]). Marathon race performance generally slows as temperature increases (Gasparetto and Nesseler, 2020[38]; Weiss et al., 2022[117]; El Helou et al., 2012[31]), with slower runners exhibiting a disproportionately greater decline in running speed under warmer conditions (Ely et al., 2007[32]). Nonetheless, even top-ten finishers demonstrate measurable performance decrements as temperatures rise (Gasparetto and Nesseler, 2020[38]; Weiss et al., 2022[117]; Llewellyn and Maceri, 2025[65]). Under identical thermal exposure, the fastest runners experienced a larger relative decline in performance than slower runners, suggesting a heightened sensitivity to heat among elite competitors (Gasparetto and Nesseler, 2020[38]).

Responses to other environmental variables also differed by performance tier. Elite runners tended to run faster with increasing humidity and longer sunshine duration, whereas the broader field of runners showed slower race times with rising temperature, humidity, and sunshine duration (Weiss et al., 2022[117]).

#### Sex

Marathon race times increased with rising temperatures, and this temperature-related performance decline did not differ between women and men across both faster and slower performance groups (Ely et al., 2007[32]). Higher humidity levels were associated with faster running speeds in both sexes (Weiss et al., 2022[116]). However, sex-specific differences emerged for other environmental variables: men were significantly more affected by variations in wind speed and humidity than women, but not by temperature. Consequently, men appeared to benefit more from higher humidity and increased wind speed than women (Knechtle et al., 2021[51]).

### Pacing in the 'New York City Marathon'

Pacing refers to the distribution of effort across a race and reflects the interaction of individual characteristics, environmental conditions, and task-specific demands (Ramos-Campo et al., 2026[89]). Recent work suggests that optimal pacing is shaped by race parameters such as distance and target speed (De Lucia et al., 2025[26]) and may support improved thermoregulation and metabolic efficiency, including energy conservation and glycogen sparing (Grivas, 2025[40]). In marathon running, pacing strategies are commonly categorized as positive, negative, or even, depending on whether speed progressively decreases, increases, or remains stable throughout the race (Sha et al., 2024[97]).

Pacing behavior in the 'New York City Marathon' has been examined in several studies (Díaz et al., 2019[27]; Santos-Lozano et al., 2014[92]; Breen et al., 2018[11]; Nikolaidis and Knechtle, 2017[82], 2018[83], 2019[81]; Lin and Meng, 2018[62]; Emerson and Manns, 2024[33]; Thuany et al., 2025[105]). Some analyses focused exclusively on this event (Santos-Lozano et al., 2014[92]; Breen et al., 2018[11]; Nikolaidis and Knechtle, 2017[82], 2018[83], 2019[81]; Birk et al., 2019[7]), whereas others combined 'New York City Marathon' data with results from additional marathons (Lin and Meng, 2018[62]; Emerson and Manns, 2024[33]).

Across studies, a general pattern of positive pacing was observed, characterized by a progressive decline in running speed over successive race segments (Santos-Lozano et al., 2014[92]; Nikolaidis and Knechtle, 2018[83]), often followed by a final acceleration-or 'end spurt'-in the last segment (40-42.2 km) (Nikolaidis and Knechtle, 2018[83]). The fastest running speeds were typically recorded between 5 and 10 km in the race, while the slowest occurred between 35 and 40 km (Nikolaidis and Knechtle, 2017[82], 2019[81]).

#### 5 km splits versus splits in miles

Most studies examining pacing in the 'New York City Marathon' have relied on 5-km split times to characterize changes in running speed across the race (Díaz et al., 2019[27]; Santos-Lozano et al., 2014[92]; Breen et al., 2018[11]; Nikolaidis and Knechtle, 2017[82], 2018[83], 2019[81]; Lin and Meng, 2018[62]). Only one study analyzed pacing using split times recorded for each mile of the course (Thuany et al., 2025[105]). In that analysis, performance in the final mile was strongly correlated with performance in the earliest mile examined (the 4^th^ mile), independent of an athlete's overall ranking position (Thuany et al., 2025[105]).

#### Age

Age influences pacing strategy in the 'New York City Marathon' (Breen et al., 2018[11]). Studies examining age-group marathoners reported a significant split × age-group interaction for running speed in both women and men (Nikolaidis and Knechtle, 2018[83]). An age-group × performance-group interaction for Δ running speed was observed across multiple race segments (5, 10, 15, 20, 25, 30, 35, and 40 km), with older athletes generally maintaining a more even running speed than younger athletes-a pattern that was especially pronounced among slower performance groups (Nikolaidis and Knechtle, 2017[82]). Consequently, fast master runners appear to adopt pacing strategies similar to those of younger fast runners (Nikolaidis and Knechtle, 2019[81]).

#### Sex

Sex influences pacing strategy in the 'New York City Marathon' (Santos-Lozano et al., 2014[92]; Breen et al., 2018[11]). Several studies have examined sex-specific pacing patterns, with one analysis reporting that men exhibited a larger decline in running speed from their fastest segment (5-10 km) to their slowest (35-40 km) compared with women (21.1 % vs. 16.7 %) (Nikolaidis and Knechtle, 2018[83]). A distinct pattern was also observed in the 25-30 km segment, where women showed an increase in speed while men slowed down (Nikolaidis and Knechtle, 2018[83]). Another study found that women demonstrated a smaller overall pace range, indicating more consistent pacing across the race (Breen et al., 2018[11]).

An additional factor is the mixed-sex race environment. As men overtake women, the performance of female runners declines differentially across ability levels, with the greatest declines occurring among lower-ability women (Birk et al., 2019[7]). Despite these sex-specific dynamics, both women and men generally aim to maintain an even pacing profile throughout the marathon, in part by avoiding an excessively fast start that could lead to a pronounced slowdown in the second half of the race (Santos-Lozano et al., 2014[92]).

#### Performance level

Performance level is a key determinant of pacing strategy in the 'New York City Marathon' (Santos-Lozano et al., 2014[92]; Breen et al., 2018[11]; Nikolaidis and Knechtle, 2019[81]; Emerson and Manns, 2024[33]). Top runners demonstrated markedly lower variability in running speed across the race, with coefficients of variation (CV) of 7.8 % for men and 6.6 % for women during 5-km splits, compared with substantially higher CV values (8.3 % to 14.4 %) among less successful runners (Santos-Lozano et al., 2014[92]).

When pacing was analyzed by performance groups, runners in the slowest group showed the largest percentage decreases in running speed at 5, 10, 15, and 20 km, but the largest percentage increases at 35 and 40 km. In contrast, runners in the fastest group exhibited the smallest decreases throughout the race and the smallest increase at 40 km (Nikolaidis and Knechtle, 2019[81]).

High-performing master athletes also demonstrated more controlled pacing strategies than their lower-ranked counterparts (Breen et al., 2018[11]). Overall, high-performing athletes displayed the narrowest pace range, with pace variability increasing progressively as performance level declined (Breen et al., 2018[11]).

An additional factor influencing pacing is the use of professional pacers. Their presence has been shown to improve race times among the fastest elite runners, although it may also reduce the perceived competitiveness of the race by creating greater separation between athletes earlier in the event (Emerson and Manns, 2024[33]).

#### Environmental conditions

Environmental conditions also affect pacing behavior in the 'New York City Marathon', with temperature and humidity exerting age-dependent effects (Weiss et al., 2022[116]). Increasing temperatures slowed runners of both sexes aged 20-59 years, whereas increasing humidity was associated with slower running speeds in athletes younger than 20 and older than 80 years (Weiss et al., 2022[116]). Ambient temperature emerged as the strongest environmental predictor of race performance, with a disproportionately negative impact on mid-to-older age group runners (Weiss et al., 2022[116]). These findings suggest that master athletes may be particularly susceptible to thermal stress during mass-participation events.

#### Nationality

Nationality also appears to influence pacing strategy in the 'New York City Marathon' (Aschmann et al., 2018[3]). Ethiopian and Kenyan runners demonstrated more even pacing profiles than athletes from other countries (Aschmann et al., 2018[3]). The prevalence of an end spurt varied substantially by nationality. American women and men showed the highest rates (87.2 % and 78.2 %, respectively), whereas Ethiopian (54.3 %) and Kenyan runners (45.8 %) exhibited the lowest prevalence of end-race accelerations (Aschmann et al., 2018[3]).

### Specific aspects of the 'New York City Marathon'

#### The bridge

A small number of studies have examined the role of the Verrazzano-Narrows Bridge in the context of the 'New York City Marathon' (Setareh, 2011[96]; Akat, 2016[2]). The Verrazzano-Narrows Bridge is a double-decked suspension bridge connecting Staten Island and Brooklyn across the Narrows. Although closed to bicycles and pedestrians under normal conditions, it has served as the starting point of the 'New York City Marathon' since 1976. Its total length of 4,176 m (13,700 ft) includes the 1,310 m (4,300 ft) approach spans on the Brooklyn side and the ⁓1,100 m (3,600 ft) approach on Staten Island.

A key aspect investigated in these studies is the structural vibration induced by thousands of runners crossing the bridge simultaneously at varying speeds (Akat, 2016[2]). Measurements of bridge motion during the marathon demonstrated that vibration levels remained within acceptable limits for both structural integrity and runner safety (Setareh, 2011[96]).

#### Disabled athletes

The 'New York City Marathon' also includes competition for disabled athletes, notably wheelchair and handcycle divisions (Lepers et al., 2014[60]). Women represented approximately 20 % of finishers in both categories (Lepers et al., 2014[60]). For both sexes, the age of the winners and the mean age of all finishers were significantly lower among wheelchair athletes compared with handcycle athletes (Lepers et al., 2014[60]). Among men, race times were significantly faster for handcycle winners and overall finishers than for their wheelchair counterparts (Lepers et al., 2014[60]). Since 2003, the sex difference in performance among wheelchair athletes has stabilized at ⁓25 %, whereas the sex difference in handcycle performance has been more variable, ranging from 15 % to 45 % (Lepers et al., 2014[60]).

#### Advanced shoe technology

Advanced running-shoe technology has been shown to improve running economy at speeds commonly used by recreational runners (Van Hooren et al., 2025[112]). One notable trend is the increasing use of toe-spring elevation, with shoes incorporating this feature exerting less pressure on the hallux during propulsion (Li et al., 2026[61]). Among world-class male marathoners, an analysis comparing advanced footwear technology with traditional shoes demonstrated a performance advantage of ⁓1 %, equivalent to ⁓79 second (Langley and Langley, 2024[56]).

In the New 'York City Marathon', one study specifically examined the impact of technologically advanced racing shoes on marathon performance (Senefeld et al., 2021[95]). Marathon finishing times were substantially faster when world-class athletes-particularly women-wore advanced racing shoes. For men, wearing neoteric Nike models was associated with a ⁓2.0 % improvement, corresponding to a ⁓2.8-min reduction in finishing time. For women, the improvement was ⁓2.6 %, or ⁓4.3 min faster compared with another footwear (Senefeld et al., 2021[95]).

#### Tourism and economy

The large influx of athletes travelling to New York for the race generates substantial demand for hotel accommodations, particularly among international and out-of-state participants (Martin and Hall, 2020[69]). As the largest competitive running event in the world, the 'New York City Marathon' attracts a broad spectrum of domestic and international athletes as well as spectators, amplifying its economic footprint across the city's hospitality, retail, and transportation sectors (Martin and Hall, 2020[69]; Montaruli et al., 2009[73]). However, this economic benefit is accompanied by a considerable environmental cost. Marathon participation is associated with a notable carbon footprint, largely driven by long-distance travel, with an estimated 37 % of 'New York City Marathon' participants flying internationally to attend the event (Castaignède et al., 2021[16]).

#### Media coverage

As one of the two largest marathon events worldwide-alongside the 'Berlin Marathon'-the 'New York City Marathon' receives extensive media attention across traditional and digital platforms (Clemons and Bogina, 2024[20]; Marxer and Tschudin, 2017[70]), including substantial engagement on social media (Park et al., 2021[88]; Boutet et al., 2016[9]). Despite this broad visibility, notable disparities exist in the coverage of different competitive divisions. Able-bodied men receive the majority of broadcast clock time in the 'New York City Marathon', a pattern more pronounced than in other major U.S. races such as the marathons in Boston and Chicago (Clemons and Bogina, 2024[20]). Coverage of both female and male wheelchair athletes is markedly limited, with the 'New York City Marathon' showing particularly low representation of these divisions (Clemons and Bogina, 2024[20]). In contrast, women receive more announcer mentions than men, often with a stronger emphasis on personal background, coaching relationships, and emotional narratives (Clemons and Bogina, 2024[20]).

#### Case studies and case reports of runners with medical conditions

Within the 'New York City Marathon', several case studies and case reports have documented exceptional athletes (Tjelta et al. 2014[106]) as well as participants with a range of medical conditions, including cystic fibrosis (Stanghelle and Skyberg, 1988[103]; Stanghelle et al., 1988[102], 2000[101]; Ceder et al., 1988[17]), type 1 diabetes mellitus (Kjeldby, 1997[47]), and congenital agenesis of the *tensor fascia lata* (Bellini et al., 2025[6]). Kjeldby published a brief report describing the experience of running the New York City Marathon while managing diabetes. The author highlights the practical challenges of maintaining stable blood glucose during prolonged endurance exercise and the strategies used, such as frequent monitoring, carbohydrate intake adjustments, and careful insulin management (Kjeldby, 1997[47]). The central message for future marathoners with type 1 diabetes mellitus is the preparation, monitoring, and understanding of one's own metabolic responses, people with diabetes can safely participate in demanding endurance events like a marathon (Kjeldby, 1997[47]). In one report, a 25-year-old male marathon finisher was incidentally found to have congenital agenesis of the *tensor fascia lata* during a muscle MRI performed for a genetic myopathy study, in which he served as a healthy control (Bellini et al., 2025[6]). Multiple reports describe finishers with cystic fibrosis, demonstrating that individuals with this condition may safely engage in strenuous, prolonged endurance exercise such as marathon running-even under hot and humid environmental conditions-without adverse effects (Stanghelle and Skyberg, 1988[103]; Stanghelle et al., 1988[102]). One particularly notable case involved a 32-year-old man who had undergone bilateral lung transplantation for end-stage cystic fibrosis 15 months prior to the event; he successfully completed the marathon in 7:08:50 (h:min:s) (Stanghelle et al., 2000[101]).

#### Case study: Grete Waitz

One case study examined the training preparation of nine-time 'New York City Marathon' champion Grete Waitz (Tjelta et al. 2014[106]). During her peak competitive seasons, her weekly running volume ranged from 119 to 132 km across different meso-cycles of the training year-substantially lower than the volumes typically reported for contemporary female world-class marathon runners. Her training structure was characterized by two daily sessions of continuous running lasting 50-60 min, performed at relatively high intensity. She completed very few long interval sessions; instead, her program generally included one weekly high-intensity workout consisting of short intervals or sprint training (Tjelta et al. 2014[106]).

#### Energy-efficient wearable analysis for running

One study introduced a portable, high-precision kinematic analysis system based on miniature MEMS motion sensors, deployed during the 'New York City Marathon' in both elite and recreational runners (Liu et al., 2017[63]). The system, known as *Gazelle*, is a compact, lightweight, and highly accurate wearable platform designed for runners across all performance levels. Its architecture incorporates Sparse Adaptive Sensing (SAS), enabling more than 200 days of continuous, high-precision data collection powered solely by a coin-cell battery, thereby offering an exceptionally energy-efficient solution for large-scale, real-world gait analysis.

#### The critical velocity test

One study compared performance outcomes from the critical velocity (CV) test with race times from the 'New York City Marathon' and found that the CV test may serve as an attractive field-based method for assessing marathon performance potential (Florence and Weir, 1997[34]). Marathon finishing time showed a stronger correlation with CV than with either VO_2_peak or ventilatory threshold (Thvent) (Florence and Weir, 1997[34]), suggesting that CV may provide a more robust indicator of sustained endurance capacity relevant to marathon performance.

## Discussion

**Figure 3:**
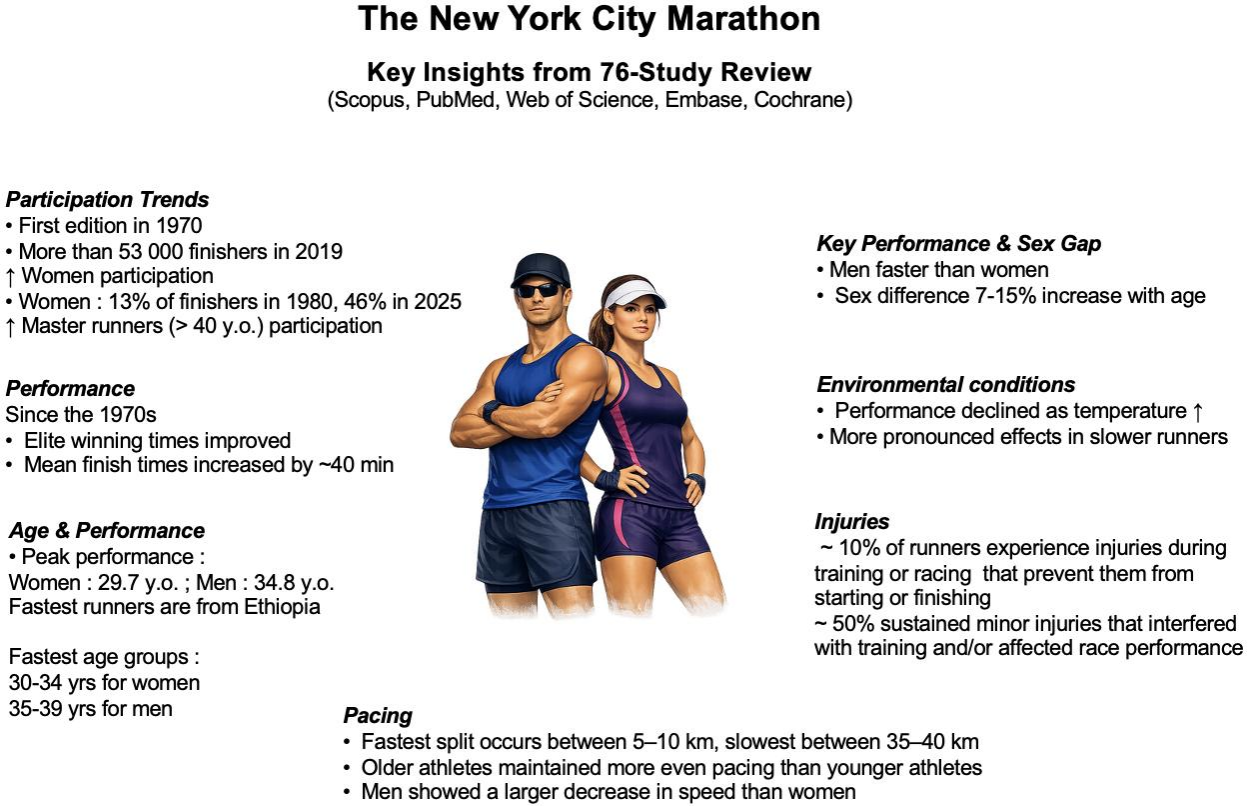
Main findings of the systematic review

The aim of this review was to comprehensively summarize and critically synthesize scientific evidence derived from studies conducted on the 'New York City Marathon', given its unique status as the world's largest mass-participation marathon and its long-standing relevance in endurance running research. We hypothesized that, despite the heterogeneity of study designs and research domains, consistent patterns would emerge across participation trends, performance, pacing, aging, injuries, and environmental influences, allowing for an integrated interpretation of how athletes perform and adapt in this specific race context. Overall, the main findings support this hypothesis (Figure 3(Fig. 3)).

Despite substantial heterogeneity across study designs, several recurring patterns were consistently reported across independent studies, and the accumulated evidence demonstrates coherent and reproducible trends, including a marked increase in participation (particularly among women and master runners), long-term improvements in elite and age group performance, and distinct sex- and age-related differences in pacing and peak performance. Additionally, a substantial burden of training- and race-related injuries and a pronounced influence of environmental factors, especially ambient temperature, on race outcomes were reported in the literature.

### Participation and performance trends in the 'New York City Marathon'

Since its inaugural edition in 1970, participation in the 'New York City Marathon' has increased steadily, with particularly pronounced growth among women and age-group runners. Similar developments have been documented in other major city marathons, including 'Berlin Marathon' (Reusser et al., 2021[90]) and 'Boston Marathon' (Knechtle et al., 2018[50], 2020[49]). These observations reflect broader trends in endurance sports participation and should be interpreted as contextual insights rather than direct evidence derived exclusively from 'New York City Marathon' data.

Over recent decades, the event has reached record participation levels, with master athletes contributing disproportionately to this rise. Comparable patterns have been observed in other endurance disciplines such as inline skating (Teutsch et al., 2013[104]) and ultra-marathon running (Knechtle et al., 2020[52]). The growing number of age-group participants may be attributed to increased health awareness, as aging populations place greater emphasis on cardiovascular fitness, weight management, and psychological well-being. Marathon running offers a structured and accessible avenue for maintaining physical activity (Cui and Zhou, 2025[24]), while large city marathons additionally provide social engagement, community belonging, and goal-oriented participation-factors particularly valued by master runners (Springer et al., 2022[100]).

Comparative data from the 'Berlin Marathon' show participation increases across nearly all age groups, except for men aged 20-49 years and athletes of both sexes over 79 years (Reusser et al., 2021[90]). Although participation remains higher among men, the relative increase has been more pronounced among women. Performance trends in 'Berlin Marathon' indicate that while average performance within age groups has declined over time, the top ten athletes in each age category have improved, suggesting simultaneous mass-participation expansion and performance gains among the highest-performing age-group runners (Reusser et al., 2021[90]).

Marathons have evolved from niche competitive events to widely celebrated mass-participation experiences. Motivations have shifted toward psychological, health-related, and social factors rather than purely competitive goals (Malchrowicz-Mośko et al., 2020[68]; Waśkiewicz et al., 2019[114]; Gómez et al., 2022[39]). Since the formal inclusion of women in major competitions in the 1970s and 1980s (e.g., 'Boston Marathon' 1972; Olympic Marathon 1984), women's participation has risen steadily, driven by changing societal norms, increased visibility of female athletes, and broader acceptance of women in endurance sports (Waśkiewicz et al., 2019[114]). Longer life expectancy, delayed retirement, and the popularity of master-level competitions have further contributed to increased participation among adults aged 30 and older (Brilliant et al., 2021[12]; Liu et al., 2025[64]). Public health campaigns promoting physical activity and cardiovascular fitness have reinforced these trends across all age groups (Brilliant et al., 2021[12]; Liu et al., 2025[64]).

Overall participation growth has been accompanied by a marked decrease in the men-to-women ratio, reflecting faster relative increases in female participation. This pattern is consistent with trends observed in other endurance and ultra-endurance sports, including trail running (Le Mat et al., 2023[58]) and additional endurance disciplines (Owen et al., 2025[86]; Buehler et al., 2026[13]). The declining ratio arises because women-historically underrepresented-are entering these events at a proportionally higher rate than men (Kong et al., 2020[55]). Although absolute male participation may also rise, the slower relative growth mathematically results in a lower men-to-women ratio. Similar dynamics are observed in labor force participation, where reductions in structural barriers lead to disproportionately higher growth among previously underrepresented groups (Dolan et al., 2023[28]).

Regarding performance, both elite and master runners of both sexes have improved over successive editions of the 'New York City Marathon'. However, average finishing times have increased over the years, reflecting the broadening participation base. This apparent paradox mirrors findings from the 'Berlin Marathon', where overall age group performance declined while the top ten athletes within each age category improved (Reusser et al., 2021[90]). Together, these trends illustrate the dual evolution of marathons as both elite sporting competitions and inclusive mass-participation events.

### Nationality aspect

Analyses of nationality-related differences in marathon performance show that Ethiopian runners-both women and men-tend to be the youngest and fastest, whereas Japanese runners are typically older and slower. When examining the annual top 100 women and men across four World Marathon Majors (Boston, Berlin, Chicago, and New York) and the 'Stockholm Marathon', age increased over time among Ethiopian men but not among Ethiopian women (Knechtle et al., 2017[48]). Ethiopian and Kenyan athletes also demonstrated more even pacing strategies than runners from other nationalities, although direct comparisons with additional studies remain limited.

Ethiopian and Kenyan marathoners consistently rank among the fastest and youngest globally, a pattern likely reflecting a combination of intrinsic physiological characteristics, altitude-acclimated development, early-life endurance exposure, and socio-cultural factors that promote competitive running (Grivas et al., 2024[41]). In contrast, Japanese marathoners-particularly in ultra-endurance events-tend to be older and slower, largely due to later entry into competitive running, differing training emphases, and event-specific participation patterns (Tokudome et al., 2004[107]). Notably, Japanese athletes are among the fastest performers in ultra-marathon distances (Cejka et al., 2014[18]). These interpretations should be applied cautiously, as much of the supporting evidence originates from contexts outside the 'New York City Marathon'.

Japanese runners typically train within a highly structured domestic system centered around university *Ekiden* relay races and corporate team competitions, which emphasize endurance, discipline, and consistent pacing rather than aggressive tactical surges (Tokudome et al., 2004[107]). In contrast, Kenyan and Ethiopian athletes frequently train and compete internationally within highly competitive circuits that demand adaptability to variable race dynamics and fast early paces. This exposure fosters tactical versatility and contributes to their success in major global marathons.

Pacing strategies also differ markedly between nationalities. Japanese marathoners often adopt conservative, steady pacing aimed at endurance preservation (Tokudome et al., 2004[107]), whereas East African runners tend to employ more aggressive, variable pacing with early surges that challenge competitors and contribute to faster overall times. These contrasting tactical approaches shape race outcomes and help explain nationality-specific performance profiles on the world stage.

### Age aspects in the 'New York City Marathon'

In the 'New York City Marathon', the fastest performances are typically achieved by runners aged 25-34 years. Depending on whether 5-year or 1-year age intervals are used, the fastest men range between 28.3 and 34.8 years of age, while the fastest women fall between 29.7 and 30.8 years.

Comparable findings have been reported in the 'Berlin Marathon', where race times show a significant positive correlation with age-indicating that older runners are generally slower than younger runners-with the association being stronger in men than in women (Knechtle et al., 2021[53]). The age of peak marathon performance in 'Berlin Marathon' was 32 years for women and 34 years for men when using 1-year age groups, and 30-34 years for women and 35-39 years for men when using 5-year age groups (Nikolaidis et al., 2019[80]).

These findings collectively suggest that peak marathon performance occurs in the early to mid-30s for both sexes, with slight variations depending on analytical resolution and event-specific characteristics.

### Injuries before and during the 'New York City Marathon'

A substantial body of research has examined running-related injuries occurring during preparation for, or participation in, the 'New York City Marathon'. Given the scale of the event, its variable course profile, and the often unpredictable environmental conditions, injury patterns observed in this race may differ from those reported in flatter or more homogeneous marathon settings. Many runners experience musculoskeletal injuries during training, which can impair performance or limit participation (Braschler et al., 2025[10]).

Chronic injury as a risk factor has not yet been systematically investigated in the context of the 'New York City Marathon'. The most relevant evidence comes from the 'Comrades Marathon', where a large cohort study of 10,973 ultra-marathon entrants identified chronic injury-alongside sex, training characteristics, and chronic disease history-as a significant factor associated with exercise-associated muscle cramping (Macmillan et al., 2024[66]). This finding highlights an important but underexplored dimension of injury-related risk in endurance events.

Comparable analyses are currently lacking for the 'New York City Marathon', underscoring the need for future research on how chronic injury history influences performance, pacing, and race-day outcomes in large city marathons.

### Cardiovascular system of 'New York City Marathon' runners

Only a limited number of studies have examined cardiovascular responses in participants of the 'New York City Marathon'. Existing work has primarily focused on changes in plasma lipid profiles before and after the race, while no studies to date have evaluated cardiac biomarkers, electrocardiographic changes, or echocardiographic parameters in this specific event. This stands in contrast to the extensive cardiovascular monitoring literature available from other major marathons, such as the 'Berlin Marathon', where biomarkers, ECG data, and imaging findings have been more comprehensively documented (Schacht et al., 2011[94], 2012[93]; Herm et al., 2017[43]; Haeusler et al., 2012[42]; Spethmann et al., 2014[99]).

Recent technological developments have demonstrated the feasibility of continuous cardiac monitoring during the 'Berlin Marathon'. Using a Holter tele-ECG system paired with a standard smartphone via Bluetooth, runners can record a full ECG throughout the marathon (Spethmann et al., 2014[101]). Real-time ECG streaming enables the detection of arrhythmias with high fidelity, with studies reporting that 100 % of rhythm disturbances can be identified in streamed ECG data (Spethmann et al., 2014[101]). These advances highlight the potential for large-scale, real-world cardiovascular surveillance in mass-participation endurance events, although such approaches remain underutilized in the context of the 'New York City Marathon'.

### Environmental conditions and race performance in the 'New York City Marathon'

Temperature appears to be the most influential environmental factor affecting marathon performance in the 'New York City Marathon', whereas humidity, dew point, and atmospheric pressure show little to no meaningful impact. These seemingly divergent findings likely reflect differences in performance level, thermoregulatory capacity, and competitive race dynamics between elite and recreational runners.

A consistent observation across studies is that marathon race times slow as ambient temperature increases. Similar associations have been reported in the 'Berlin Marathon', where higher air temperatures correlated with slower finishing times among both average and elite male runners (El Helou et al., 2012[31]; Llewellyn and Maceri, 2025[65]; Knechtle et al., 2021[54][53]; Weiss et al., 2022[118]).

Rising temperatures disproportionately impair performance in slower runners, although even top-ten finishers tend to slow down under hotter conditions. In the 'Berlin Marathon', faster runners experienced lower temperatures simply because they completed the race earlier in the day, before temperatures rose to levels encountered by slower participants (Weiss et al., 2022[118]). Among age-group runners, a negative correlation has been reported between increasing temperature, declining humidity throughout the day, and running speed, with this effect being more pronounced in men than in women (Weiss et al., 2024[115]).

Overall, runners tend to slow with increasing temperature and sunshine duration. However, an exception has been noted among elite athletes: top-three and top-ten finishers in some analyses improved their race times as temperatures increased, with women showing greater improvements than men (Knechtle et al., 2021[53]). This pattern may reflect differences in pacing strategies, heat tolerance, and competitive dynamics at the elite level.

### Pacing in the 'New York City Marathon'

Analyses of pacing patterns in the 'New York City Marathon' show a characteristic positive pacing profile, with running speed gradually declining over the race and a final acceleration in the last segment (40-42.2 km). The fastest split typically occurs between 5-10 km, while the slowest is observed between 35-40 km.

Age-related differences in pacing: Older age-group runners tend to maintain a more even pace than younger athletes. Comparable findings were reported in the 'Oslo Marathon', where pace variability was greatest in the youngest and oldest runners (12.55 % vs. 10.96 %) (Cuk et al., 2021[25]). This suggests that middle-aged runners may adopt more efficient pacing strategies, possibly due to greater experience or more conservative race execution.

Sex differences in pacing: Men generally exhibit larger reductions in running speed across the marathon than women. In the 'New York City Marathon', men showed a 21.1 % decrease from their fastest (5-10 km) to slowest (35-40 km) split, compared with 16.7 % in women. Interestingly, at 25-30 km, women increased their speed slightly, whereas men slowed. Findings from the 'Berlin Marathon' support these patterns. Male runners who adopted even pacing achieved significantly faster performances than those using positive pacing strategies (Muñoz-Pérez et al., 2023[75]). Among men, pacing differences across performance groups were minimal, whereas women showed numerous significant differences based on performance level (Muñoz-Pérez et al., 2020[76]). When pacing behavior was analyzed across performance groups, no significant split-to-group differences were found in men, but many were observed in women (Muñoz-Pérez et al., 2023[75]). Overall, women reduced their running speed less than men in the second half of the marathon, particularly after 35 km (Muñoz-Pérez et al., 2023[75]).

Pacing and performance level: Performance level strongly influences pacing strategy. The slowest runners show the largest percentage decreases in speed at 5, 10, 15, and 20 km, but the greatest increases at 35 and 40 km-likely reflecting a late-race effort to maintain or regain pace. In contrast, the fastest runners exhibit the smallest speed reductions throughout the race and the smallest late-race increases. A study comparing performance tiers (world record holders; champions; 2^nd^-5^th^ place; 6^th^-20^th^ place) found that world-record men used a constant pacing strategy. Both male and female champions also maintained constant pacing, whereas the 2^nd^-5^th^ and 6^th^-20^th^ place groups of both sexes adopted positive pacing strategies. No differences in pacing strategy were observed between winning men and women (Oliveira et al., 2022[85]).

### Why is this specific marathon uniquely informative compared to other major marathons?

The 'New York City Marathon' provides an unusually rich setting for understanding endurance performance, mass-event logistics, and medical risk because it combines extreme participant diversity, complex urban geography, and substantial environmental variability. Unlike flatter, more uniform World Marathon Majors, the 'New York City Marathon' functions as a natural experiment in how athletes and support systems respond to heterogeneous stressors across an entire megacity.

The five-borough route exposes runners to rapid shifts in terrain, wind exposure, crowd density, and microclimate. Bridges introduce repeated incline-decline cycles and strong crosswinds, while long stretches of sun-exposed roadway alternate with shaded avenues (Franklin, 2026[35]). This variability produces measurable fluctuations in thermoregulatory load, cardiovascular strain, and pacing stability. In contrast, races such as 'Berlin Marathon' or 'Chicago Marathon'-designed for uniformity and speed-offer limited insight into how athletes adapt to changing environmental demands (World Academy for Endurance Medicine, 2026[120]).

The course of the 'New York City Marathon' also generates predictable pacing disruptions at bottlenecks and on bridges, allowing researchers to study how external constraints shape energy expenditure, fatigue accumulation, and risk of collapse. These dynamics are difficult to observe in more homogeneous courses (Futterman, 2026[36]).

With more than 50,000 finishers annually, the 'New York City Marathon' provides the largest and most demographically diverse dataset in marathon running. The field includes a broad distribution of ages, performance levels, and international participants, enabling analyses that would be underpowered elsewhere (Dutch and Boswell, 2026[30]). Rare but clinically important events-such as exertional collapse, exercise-associated hyponatremia, or heat-related illness-occur frequently enough to support robust epidemiological modeling. This scale also allows examination of how travel, jet lag, pre-race routines, and differing training backgrounds influence outcomes. The resulting insights extend beyond elite performance to the realities of mass-participation endurance sport.

The multi-wave start system, long transport routes, and extended waiting times at the start village create 2-4 hours of pre-race exposure to cold, wind, or heat. These conditions influence hydration behavior, core temperature, and early-race pacing. Few other major marathons impose such prolonged pre-race environmental load, making the 'New York City Marathon' uniquely suited to studying how anticipatory stress affects performance and medical risk.

New York's early-November timing has produced marathons run in unseasonable heat, cold snaps, and high winds. This inter-annual variability enables comparisons of heat-related illness, dropout rates, and pacing deterioration across different environmental conditions. The 'New York City Marathon' therefore serves as a real-world testbed for validating weather-based risk thresholds and for understanding how environmental stress interacts with course design and participant characteristics (Weiss et al., 2022[116]).

The 'New York City Marathon' requires coordination across dozens of medical stations, thousands of volunteers, multiple hospital systems, and emergency services spanning five boroughs (crowdRX, 2026[23]). This complexity generates insights into triage patterns, resource allocation, and communication strategies that are directly transferable to other mass-participation events. Analyses of medical encounters along the course help identify high-risk segments, refine triage algorithms, and optimize the placement of medical resources (New York Road Runners, 2026[79]). Athletes benefit from evidence-based pacing strategies tailored to the course's variable demands, improved hydration and nutrition planning informed by pre-race waiting times, and better preparation for a wide range of environmental conditions. Organizers gain data to refine wave starts, manage crowd flow, and position medical resources more effectively (World Marathoner, 2026[121]). Insights into pre-race stress and congestion support improved communication and logistical planning. Medical teams can develop predictive models for collapse and heat illness, refine triage protocols based on high-volume encounter data, and design scalable emergency response systems applicable to other large events (New York Road Runners, 2026[79]).

The 'New York City Marathon' thus serves as a uniquely informative platform for understanding endurance physiology, mass-event logistics, and medical preparedness under real-world conditions. Its insights extend beyond marathon running to broader questions of urban event management, public-health planning, and human performance under variable environmental stress (Wilson, 2026[119]).

### Limitations

A key limitation of the available literature is that some studies combined data from the 'New York City Marathon' with data from other marathon events. Although this approach increases sample size and enhances generalizability, it also introduces substantial heterogeneity in course profiles, environmental conditions, participant demographics, and organizational characteristics. Such variability may have influenced the reported outcomes and complicates efforts to draw conclusions that are truly specific to the 'New York City Marathon'. A future review might only limit to data obtained from the 'New York City Marathon'.

Most included studies relied on retrospective race databases, which are subject to several potential sources of error, including inaccuracies in registration data, missing or incomplete split times, and changes in timing technologies over multiple decades. These factors may affect data quality and comparability across years, thereby limiting the precision of long-term trend analyses.

### Implications for future research

Future research in the 'New York City Marathon' should prioritize the evaluation of advanced cardiovascular monitoring technologies, including the feasibility and clinical utility of real-time ECG streaming during the race. Continuous cardiac surveillance has the potential to provide unprecedented insights into arrhythmias, myocardial strain, and acute physiological responses in a large and heterogeneous population of endurance runners. Such data could help identify early warning patterns, refine on-course medical support strategies, and deepen understanding of cardiovascular stress during mass-participation events. Today, with easier access to wearables, it has become easier to control basic physiological parameters in order to manage intensity during a marathon. This can help reduce health problems, injuries, etc. as well as performance variables such as pace. Technological innovations-particularly the widespread adoption of carbon-plated 'supershoes'-represent another important avenue for investigation. Although these shoes have transformed marathon performance globally, their specific impact within the unique environmental and course characteristics of the 'New York City Marathon' remains unknown. Future studies should examine how supershoes influence training volume, running economy, pacing behavior, fatigue resistance, and injury risk in this race, and whether these effects differ by sex, age group, or performance level. Integrating wearable sensor data with machine-learning approaches offers additional opportunities to model pacing dynamics, fatigue development, and injury risk in large-scale marathon populations. Such multimodal datasets could enable more precise prediction of performance trajectories, individualized risk profiling, and the development of targeted interventions to optimize safety and performance in mass-participation marathons.

## Conclusions

The long-term trends in participation, performance, injury burden, and pacing behavior in the 'New York City Marathon' reveal how a large and increasingly diverse running population responds to the demands of endurance training and competition. The steady rise in female and recreational (age group) athletes underscores the need for training frameworks and medical guidelines that reflect the characteristics of recreational (age group) athletes rather than relying solely on elite runners. The widening gap between elite improvements and stable average race times highlights persistent disparities in pacing discipline, heat tolerance, and fatigue management-areas where targeted education and individualized training could meaningfully improve outcomes for the broader field. Age- and sex-specific performance peaks, along with specific pacing profiles, provide actionable benchmarks for tailoring periodization, goal setting, and mid-race decision-making. The high prevalence of musculoskeletal injuries, particularly among runners with large training volumes, reinforces the importance of load management, progressive conditioning, and early identification of overuse symptoms, while the protective effect of adequate preparation emphasizes the value of structured, evidence-based training plans for recreational (age group) athletes. The strong influence of ambient temperature on performance-especially among slower runners-demonstrates the need for adaptive pacing strategies, heat-risk communication, and environmental contingency planning in mass-participation events. The characteristic positive pacing pattern, with pronounced variability among slower athletes, offers insight into behavioral and physiological responses to fatigue that can inform coaching interventions and real-time decision support. Together, these findings illustrate how the 'New York City Marathon' functions as a large-scale model for understanding endurance performance under real-world constraints. They provide a foundation for developing more effective training protocols, optimizing race-day strategies across performance levels, and strengthening public-health preparedness for mass-participation endurance events.

## Declaration

### Conflict of interest

The authors declare no competing interests.

### Artificial Intelligence (AI) – assisted technology

ChatGPT was used to assist in generating and refining graphical elements included in the figures. The final design and layout were produced by the authors using Canva.

### Funding

No funding

### Acknowledgements

Not applicable

### Ethics approval and consent to participate

Not applicable

### Consent for publication

Not applicable

### Availability of data and materials

Not applicable

### Authors' contributions

Planning: Beat Knechtle; Search conduction: Beat Knechtle, Mabliny Thuany; Original draft: Beat Knechtle; Edit and critical review: Pantelis T. Nikolaidis, Pedro Forte, Luciano Bernardes Leite, Romuald Lepers, Daniela Chlíbková, Katja Weiss, Thomas Rosemann, Sasa Duric. All authors read and approved the final manuscript.

## Figures and Tables

**Table 1:**
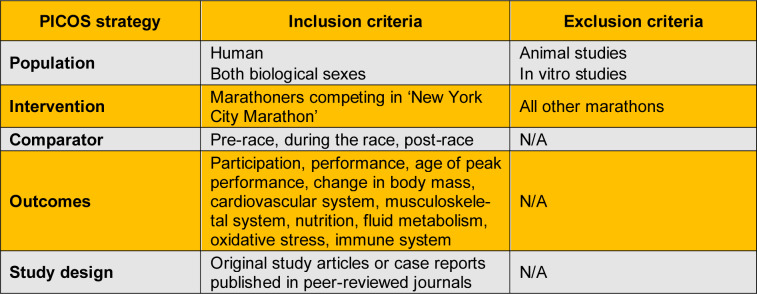
Eligibility criteria following the PICOS strategy

**Figure 1:**
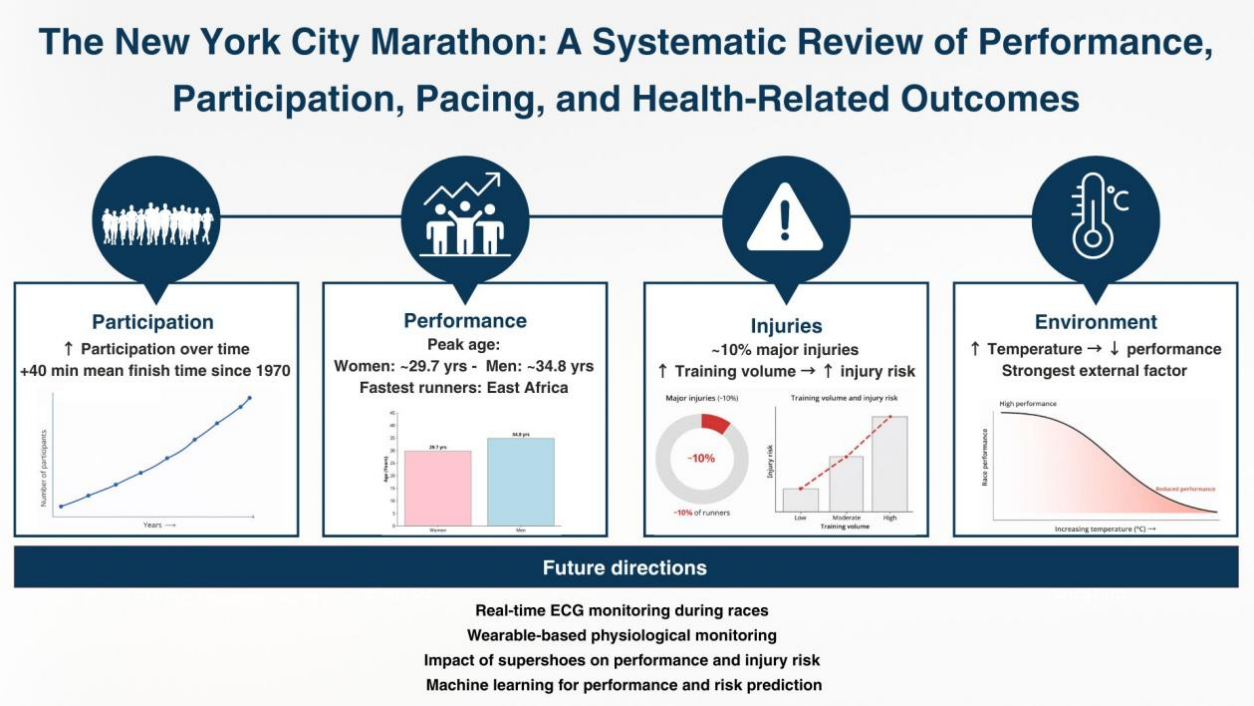
Graphical abstract
